# Interferon-Inducible Gene Upregulation Correlates With Successful Viral Clearance in Patients With BK Polyomavirus-Associated Nephropathy

**DOI:** 10.1016/j.ekir.2026.106551

**Published:** 2026-04-22

**Authors:** Haris Omic, Nicolas Kozakowski, Johannes Kläger, Hans H. Hirsch, Benjamin A. Adam, Michael Mengel, Lukas Weseslindtner, Željko Kikić, Michael Eder

**Affiliations:** 1 Division of Nephrology and Dialysis, Department of Medicine III, Medical University of Vienna, Vienna, Austria; 2 Department of Pathology, Medical University of Vienna, Vienna, Austria; 3 Transplantation and Clinical Virology, Department of Biomedicine, University of Basel, Basel, Switzerland; 4 Virus Immunity Pathology, Immunology Research, Department of Medical Biology, Faculty of Health Sciences, UiT The Arctic University of Tromsø, Tromsø, Norway; 5 Department of Laboratory Medicine and Pathology, University of Alberta, Edmonton, Alberta, Canada; 6 Center for Virology, Medical University of Vienna, Vienna, Austria; 7 Department of Urology, Medical University of Vienna, Vienna, Austria

**Keywords:** BK virus, gene expression, kidney transplantation, NanoString nCounter analysis, polyomavirus nephropathy, viral load

## Abstract

**Introduction:**

BK polyomavirus-associated nephropathy (BKPyVAN) remains a challenging complication after kidney transplantation. Although BKPyV-DNAemia is recommended for screening and reducing immunosuppression, reliable predictors of antiviral immune control are needed. The aim of this study was to identify beneficial gene expression patterns, associated with BKPyV-DNAemia clearance, using a commercially available transcriptomic array assay.

**Methods:**

We retrospectively analyzed RNA-based gene expression patterns in 30 biopsy samples with BKPyVAN, using the NanoString nCounter system. We assessed the relationship between gene expression patterns and subsequent BKPyV-DNAemia dynamics, including viral clearance rates at 6 and 12 months after BKPyVAN diagnosis, as well as the course of intermediate graft function.

**Results:**

Complete BKPyV-DNAemia clearance was observed in 40% of patients (at 6 and 12 months). BKPyV clearance was significantly associated with upregulation of interferon (IFN)- stimulated genes (ISG): *ISG15* (log fold change [logFC]: 1.84–2.02), IFN alpha–inducible protein 27 (*IFI27*) (logFC: 1.46–1.73), IFN-induced protein with tetratricopeptide repeats 1 (*IFIT1*) (logFC: 1.62–1.87), and IFN-induced transmembrane protein 1 (*IFITM1*) (logFC: 1.41–1.61). Receiver operating characteristic curve (ROC) analyses revealed areas under the curve (AUCs) of 0.80 to 0.85 for the prediction of DNAemia clearance at 6 months and 0.91 to 0.97 at 12 months (*P* < 0.01 for both). Higher ISG expression correlated with lower estimated glomerular filtration rate (eGFR) at 6 and 12 months, but not at 24 months. No significant correlations were observed with Banff lesion scores or PVN/AST-IDCOP categories.

**Discussion:**

Significant upregulation of ISGs in kidney allografts with biopsy-proven BKPyVAN was associated with successful clearance of DNAemia. Higher ISG expression correlated with temporarily worsened graft function, which stabilized on longer follow-up. This first exploratory study suggests a potential role for intragraft ISG measurement in monitoring anti-BKPyV immune response.

## Graphical abstract

**Figure:**
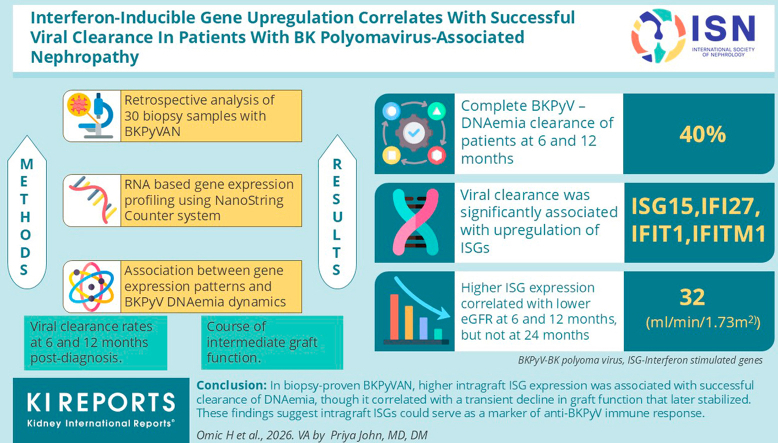

BKPyVAN remains a serious complication after renal transplantation. Because antiviral treatments are unavailable, only reducing immunosuppression is currently recommended.^1^ Subsequently, increasing BKPyV-specific immunity is thought to be the key for viral clearance; however, that increases the risk for alloimmune responses and subsequent rejection. High viral loads in plasma (BKPyV-DNAemia) are currently recommended to identify kidney transplant recipients at an early stage of disease.^2^ However, definite diagnosis of BKPyVAN requires allograft biopsy and is recommended in case of graft dysfunction or immunological high-risk patients.^1^ Nevertheless, the timing of the biopsy is critical. With incipient immune reconstitution and virus clearance, SV40 staining may be negative, despite persistent BKPyV-DNAemia.^1^^,^^3^^,^^4^ In this context, the interpretation of inflammatory cell infiltrates, responsible for successful antiviral response, may be interpreted as alloimmune-targeted cells within a T-cell–mediated rejection (TCMR), instead of resolving BKPyVAN. Intragraft gene expression profiling with the Molecular Microscope Diagnostic System or the NanoString nCounter System were suggested to solve this diagnostic uncertainty.^5^^,^^6^ To facilitate their use, the Banff Molecular Diagnostics Working Group proposed consensus gene sets, classifying the major clinical phenotypes of transplant rejection.^6^ In 2020, Adam *et al.*,^7^ analyzed intragraft gene expression profiles in BKPyVAN biopsies and revealed that virus-specific genes, such as *LTAG,* encoding the large T antigen- (LTag) or *VP1-3,* encoding the respective capsid proteins, are significantly upregulated compared to biopsies with pure TCMR. However, human molecular transcriptome patters overlapped strongly with pure TCMR samples, indicating that antiviral- and alloimmune responses do not differ significantly in their gene expression profiles.

Furthermore, diagnostic biomarkers that can predict the further course of BKPyVAN are lacking. Different histological scores have been correlated with allograft outcome of BKPyVAN^8^, ^9^, ^10^, ^11^, ^12^; however, their reproducibility was limited in subsequent studies.^13^, ^14^, ^15^, ^16^ This might be because of inherent limitations of histology, including sampling error, especially for focally distributed pathologies such as BKPyVAN.^4^

In addition, several noninvasive immune monitoring strategies, particularly BKPyV-specific T-cell assays, have gained increasing attention and have been associated with viral control.^17^, ^18^, ^19^ Although these assays provide important insights into systemic antiviral immunity, they are not yet standardized, are only available in specialized centers, and their role in routine clinical decision-making remains incompletely defined. Moreover, peripheral blood–based assays reflect systemic immune responses and may not fully capture local immune activity within the renal allograft. In this context, intragraft transcriptomic profiling offers a complementary approach by directly interrogating immune activation at the site of disease.

In a previous study, we observed that BKPyV-DNAemia dynamic was a significant predictor of allograft survival in kidney transplant recipients with biopsy-proven BKPyVAN.^15^ However, reliable allograft parameters predicting the course of BKPyV-DNAemia clearance are missing. Measuring intragraft transcriptomics simultaneously assessing a multitude of different cellular genes might offer novel insights. We hypothesized that individual genes or gene signatures that reflect antiviral immune responses predictive of BKPyV-DNAemia clearance could be identified.

To test this hypothesis, we retrospectively assessed BKPyVAN biopsies archived at our institution and correlated gene expression profiles with clinical end points. Therefore, a highly granular footprint of intragraft gene expression levels could help to identify the following: (i) inflammatory responses associated with clearing BKPyV and (ii) patients with a high risk of nonresponse.

## Methods

### Study Design and Data Collection

This retrospective cohort study analyzed gene expression profiles from adult renal allograft recipients diagnosed with biopsy-proven BKPyVAN, as defined by recent guidelines.^1^ All patients were transplanted at the Medical University of Vienna (between 2008 and 2018). Included samples originated from biopsy slides archived at the Department of Pathology, Medical University of Vienna. Inclusion of cases was based on the availability of biopsy material necessary for gene expression analysis and on a minimum clinical follow-up of period of 1 year after BKPyVAN diagnosis. Gene expression analysis was performed at 2 different University Medical Centers (the Medical University of Vienna, Austria and the University of Alberta, Canada). Fourteen biopsy samples, obtained at the Medical University of Vienna were analyzed in Edmonton, Alberta, Canada as part of an international multicenter study.^7^ The second group (16 biopsies) included samples obtained and analyzed at the Medical University of Vienna. Ethical approval was obtained from the institutional review board of the Medical University of Vienna (reference number: 1543/2018), and the study was conducted in compliance with all applicable ethical standards (Declarations of Helsinki and Istanbul).

### Primary and Secondary End Points

The main objective of this study was to identify associations between intragraft gene expression patterns and BKPyV-DNAemia clearance in patients with biopsy-proven BKPyVAN. The primary end point was viral clearance, defined as BKPyV-DNAemia below the assay’s lower limit of quantification of 100 copies/ml, limit of detection: 70 copies/ml (definition in accordance with current guidelines).^1^ BKPyV-DNAemia clearance was evaluated at 6 and at 12 months after biopsy-proven BKPyVAN (index biopsy). The applied method of BKPyV-DNAemia quantification, and the institution’s screening protocol have been described previously.^20^^,^^21^ The secondary end point was renal allograft function, determined using the Chronic Kidney Disease-Epidemiology Collaboration eGFR formula at 6 and 12 months after the index biopsy.

### BKPyVAN Diagnosis and Gene Expression Analysis

The diagnosis of BKPyVAN was based on SV40-positive immunohistochemistry and the presence of BKPyV-DNAemia. In the absence of a prespecified prospective protocol, we evaluated intragraft transcriptomic patterns at the time of clinically indicated biopsies, reflecting real-world practice. For study inclusion, all selected samples were reassessed by a nephropathologist. All included biopsies were considered diagnostically adequate at the time of evaluation by an experienced renal pathologist and sufficient for assessment of BKPyVN class and extent, in line with published recommendations.^11^ Both the PVN and the AST-IDCOP score were graded.^8^^,^^22^ Gene expression analysis was performed using the NanoString nCounter platform (NanoString Technologies, Seattle, WA). Archived formalin-fixed sections were subjected to RNA extraction using a commercially available RNA extraction kit (RNeasy FFPE; Qiagen Technologies), following the manufacturer's protocol. The concentration of isolated RNA was measured using the NanoDrop spectrophotometer. The PanCancer Immune Profiling Panel plus 30 additional custom genes, including 5 polyomavirus genes (*AGNO*, *LTAG*, *VP1*, *VP2*, and *VP3*) and 25 additional immune-related genes was used for all samples analyzed in Edmonton.^7^^,^^23^ For the second group, analyzed in Vienna, the TG Panel (including 500 genes) was used. Both panels include internal reference genes for normalization. Each assay incorporated a panel standard, consisting of a pool of synthetic DNA oligonucleotides representing the target sequences of the probe sets, facilitating consistent normalization across users, instruments, and reagent lots. Only the overlapping genes were included into the final analysis (described online at https://nanostring.com/em_resources_type/gene-probe-list/ and summarized in Supplementary Table S1). All molecular analyses were performed on index biopsies obtained at the time of initial BKPyVN diagnosis, before protocolized immunosuppression reduction.

### Statistical Tests

Statistical analysis was performed using IBM SPSS Statistics for Mac (version 29.0.2.0; IBM Corp., Armonk, NY) and R Studio (version 2024.09.1+394; Posit team, 2024; Posit Software, PBC, Boston, MA) Descriptive statistics such as mean ± SD or median (interquartile range [IQR]) were used to summarize continuous variables, whereas categorical variables were presented as frequencies and percentages. Measured expression values were normalized using the means of the supplied controls and housekeeping genes. Normalization of gene expression data was conducted using nSolver Analysis Software (version 4.0, NanoString Technologies, Seattle, WA, 2020). The analysis of the expression data was conducted using R for Mac with the previously published R Package “nanostringr.”^24^ After quality control and normalization, each gene’s expression was log_2_-transformed to stabilize the variance. Differential expression analysis was performed in R using the “limma” package. Statistical methods for differential expression analysis are described in detail in the Supplementary Methods. To control for multiple comparisons, the Benjamini–Hochberg procedure was applied, yielding false discovery rate–adjusted *P*-values. Genes with an adjusted *P*-value below a defined threshold (*P* < 0.01) were considered differentially expressed. The gene expression patterns were displayed as a volcano plot, with appropriate logFC values and their false discovery rate–adjusted *P*-values.

Genes significantly associated with the primary end point were included in additional single-gene logistic regression and ROC. Spearman correlation tests were used to assess relationships between continuous variables and gene expression levels.

To exclude significant confounders, basic clinical parameters were tested for effects on viral clearance.

The ROC analysis was used to assess the predictive power of each significant gene for viral clearance after 6 and 12 months. For the cross-validation procedure**,** we evaluated our logistic regression models using a leave-one-out cross-validation approach (Supplementary Methods). Finally, the predictive performance measures (such as AUC) are averaged across all iterations, providing an overall estimate of how well the model generalizes to unseen data. A *P*-value < 0.05 was considered significant for all analysis except differential gene expression. For viral clearance (6 and 12 months) and graft function (eGFR), we used the last observation carried forward method to align assessments with the targeted horizons. The most recent postbaseline status or value before the horizon was carried forward. In cases of graft loss at month 6, the month-6 status was carried forward to month 12 (and month 24) by design. To align assessments at 6 and 12 months, eGFR values after graft failure (or end point occurrence) were set to a fixed floor of 7 ml/min per 1.73 m^2^; no model-based imputation was used. Additional sensitivity analyses were performed to assess robustness with respect to potential confounders, including biopsy indication (indication vs. protocol), exposure to rejection or rejection treatment, and disease severity as assessed by the BKPyVAN (PVN) score.

## Results

### Baseline Characteristics and BKPyVAN Histology

Thirty adult renal allograft recipients, transplanted at the Medical University of Vienna between 2008 and 2018 were included. The mean (± SD) age at transplantation was 63.4 (± 17.4) years and 20 patients (66.7%) received a kidney from a deceased donor. Two patients (6.7%) received an ABO-incompatible organ. The immunosuppressive regimen after transplantation included mainly tacrolimus (93.4%) and mycophenolate mofetil (96.7%). One patient received cyclosporine A, 1 received sirolimus. By the end of the first posttransplant month, all patients were tapered to a maintenance dose of 5 mg prednisolone daily, according to our center’s standard immunosuppressive protocol. At baseline, 1 patient (3.3%) had positive donor-specific antibodies. Further baseline parameters are displayed in Table 1. Median time to first detection of BKPyV-DNAemia and diagnosis of biopsy-proven BKPyVAN was 2 (IQR: 2–5) months and 6 (IQR: 3–8) months after transplantation, respectively. Median BKPyV-DNAemia at the time of index biopsy was 2.6 x 10^4^ copies/ml (IQR: 7.2 x 10^2^ to 1.2 x 10^5^). At the time of BKPyVAN diagnosis, all patients were on tacrolimus-based immunosuppression. Twenty-three biopsies (77%) were clinically indicated whereas the remaining 7 patients (23%) underwent protocol biopsies in the setting of stable graft function. Twenty-four patients (80%) showed increasing BKPyV-DNAemia before biopsy. Longitudinal trajectories of BKPyV-DNAemia and eGFR relative to biopsy are shown in Supplementary Figure S1. Majority of biopsies had moderate to severe PVN scores: PVN1 (*n* = 3/10%), PVN2 (*n* = 16/53%), and PVN3 (*n* = 11/37%). AST-IDCOP classification showed PyVAN-A (mild) in 10 (30.0%), PyVAN-B (moderate) in 14 (46.7%), and PyVAN-C (advanced) in 6 (23.3%) biopsies. PyVAN-B subscores were as follows: B1 in 9 (30%) and B2 in 5 cases (16.7%).

**Table 1:** Baseline characteristics and follow-up data of the study cohort

Variable	
Recipient age at transplantation, *yrs,* mean (SD)	63 (17)
Male sex, n (%)	21 (70)
Cold ischemia time in h, median (IQR)	14 (3–17)
HLA mismatch sum, median (IQR)	2 (1–4)
Deceased donor, n (%)	20 (66.7)
ABOi transplantation	2 (6.7)
DSA positive	1 (3.3)
Valganciclovir prophylaxis	23 (77%)
Induction, n (%)	24 (80)
IL-2 receptor antibodies	17 (57)
ATG	7 (23)
Plasma exchange	5 (13)
No induction	3 (10)
Induction with rituximab	3 (10)
Time to initial BKPyV-DNAemia (mos), median (IQR)	2 (2–5)
BKPyV-DNAemia at BKPyVAN diagnosis, copies/ml, median (IQR)	2.6 × 10^4^ (7.2 × 10^2^ to 1.2 × 10^5^)
BKPyV-DNAemia 1 mo after diagnosis	9.0 × 10^4^ (1.4 × 10^4^ to 1.1×10^6^)
BKPyV-DNAemia 3 mos after diagnosis	1.6 × 10^4^ (2.6 × 10^3^ to 6.8 × 10^4^)
BKPyV-DNAemia 6 mos after diagnosis	1.4 × 10^3^ (1.0 × 10^2^ to 2.6 × 10^4^)
BKPyV-DNAemia 12 mos after diagnosis	7.9 × 10^2^ (1.0 × 10^2^ to 5.1 × 10^3^)
Maintenance IS at BKPyVAN diagnosis, n (%)	
Maintenance IS with tacrolimus	30 (100)
Maintenance IS with MMF/MPA	25 (83.3)
Maintenance IS with leflunomide	3 (10)
Maintenance IS with azathioprine	1 (3.3)
Maintenance IS with sirolimus	1 (3.3)
Tacrolimus trough levels in ng/ml, median (IQR)	
At BKPyVAN diagnosis	8.1 (6.4–12)
One mo after diagnosis	6.7 (5.8–9.4)
Three mos after diagnosis	6.5 (5.2–8.3)
Six mos after diagnosis	4.6 (3.5–6.3)
Twelve mos after diagnosis	5.8 (4.4–7.1)
eGFR (CKD-EPI), (ml/min per 1.73 m^2^), median (IQR)	
eGFR at BKPyVAN diagnosis	38.5 (30.6–44.4)
eGFR 1 mo after diagnosis	39.8 (28.9–58.0)
eGFR 3 mos after diagnosis	35.5 (30–49.9)

ABOi, ABO incompatible; ATG, antithymocyte globulin; BKPyV, BK polyomavirus; BKPyVAN, BK polyomavirus–associated nephropathy; CKD-EPI, Chronic Kidney Disease Epidemiology Collaboration (equation); DNAemia, detectable viral DNA in blood; DSA(s), donor-specific antibodies; eGFR, estimated glomerular filtration rate; HLA, human leukocyte antigen; IL-2, interleukin 2; IQR, interquartile range; IS, immunosuppression; MMF, mycophenolate mofetil; MPA, mycophenolic acid.

Concomitant antibody-mediated rejection was diagnosed in 2 cases. Six patients received glucocorticoids (500 mg i.v. aprednislone for 3 consecutive days) and 2 patients received anti-thymocyte globulin for initially clinically assumed TCMR (following the biopsy). One patient with concomitant antibody-mediated rejection underwent plasmapheresis. The distribution of Banff lesions and biopsy adequacy parameters is provided in Supplementary Table S2. No patient had glomerulitis lesions. No patient was lost to follow-up. Four patients (13.3%) lost their allograft within 12 months (one of them before month 6).

### Gene Expression Analysis and Viral Clearance

**Figure 1:**
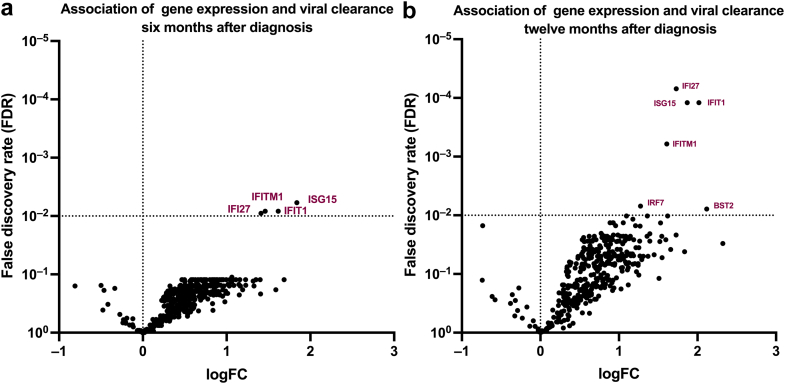
Gene expression analysis compared between patients with and without viral clearance 6 months (a) and 12 months (b) after the histological diagnosis. LogFC are presented on the x-axis, and *P* values for FDR on the y-axis. The dotted line represents a significance level of 0.01 for FDR. For visualization purposes, only the genes with significant levels are illustrated and annotated in color. *IFI27*, IFN alpha–inducible protein 27; *IFIT1*, IFN-induced protein with tetratricopeptide repeats 1; *IFITM1*, IFN-induced transmembrane protein 1; *ISG15*, interferon-stimulated gene 15; logFC, LogFold changes.

BKPyV-DNAemia clearance rates after 6 and 12 months were 40% (12 patients at each time point, because 2 patients achieved clearance after 6 months but showed low-level DNAemia at 12 months). BKPyV-DNAemia clearance after 6 months was significantly associated with upregulation of genes involved in the IFN response pathway: *ISG15* logFC: 1.84 (adjusted *P*-value < 0.01), *IFI27* logFC: 1.46 (*P* < 0.01), *IFIT1* logFC: 1.62 (*P* < 0.01), and *IFITM1* logFC: 1.41 (*P* = 0.01), as illustrated in Figure 1 (Panel a). Similar associations were found for viral clearance at month 12: *ISG15* logFC: 2.02 (*P* < 0.001), *IFI27* logFC: 1.73 (*P* < 0.001), *IFIT1* logFC: 1.87 (*P* < 0.001), *IFITM1* logFC: 1.61 (*P* < 0.001). Bone marrow stromal cell antigen 2 and IFN regulatory factor 7 showed significant association only after 12 months (logFC: 2.12, *P* = 0.007 and logFC: 1.27, *P* = 0.006, respectively, Figure 1 Panel b). All differential expression results for the analyzed genes are provided in Supplementary Table S3. A detailed description of patients with and without clearance after 12 months is depicted in Table 2.

**Table 2:** Summary of relevant clinical variables compared between patients with- and without BKPyV-DNAemia clearance after 12 mos

Variable	BKPyV-DNAemia clearance by 12 mos (n = 12)	No BKPyV-DNAemia clearance by 12 mos (n = 18)	*P*-value
Time to BKPyV-DNAemia after KTX, m, median (IQR)	3 (2–6)	2 (2–6)	0.19
Time to biopsy after KTX, m, median (IQR)	6 (3–8)	4.5 (3–6)	0.31
BKPyV-DNAemia, copies/ml, median (IQR)			
At BKPyVAN diagnosis	8.8 × 10^4^ (2.4 × 10^4^ to 5.2 × 10^5^)	4.6 × 10^3^ (1.0 × 10^2^ to 6.7 × 10^4^)	0.05
BKPyV-DNAemia 1 mo after diagnosis	3.4 × 10^5^ (3.6 × 10^4^ to 2.8 × 10^6^)	8.2 × 10^4^ (5.0 × 10^3^ to 6.5 × 10^5^)	0.10
BKPyV-DNAemia 3 mos after diagnosis	3.9 × 10^3^ (1.1 × 10^3^ to 1.9 × 10^4^)	5.6 × 10^4^ (4.8 × 10^3^ to 3.0 × 10^5^)	< 0.01
BKPyV-DNAemia 6 mos after diagnosis	1.0 × 10^2^ (1.0 × 10^2^ to 1.2 × 10^2^)	8.0 × 10^3^ (1.6 × 103 to 6.9 × 10^4^)	< 0.001
Tacrolimus trough levels in ng/ml, median (IQR)			
At BKPyVAN diagnosis	8.0 (6.6–10.4)	8.0 (6.3–12.4)	0.88
One month after BKPyVAN diagnosis	7.8 (5.9–10.9)	6.60 (5.4–8.0)	0.50
Three months after BKPyVAN diagnosis	5.9 (5.1–7.9)	6.8 (5.5–8.8)	0.30
Six months after BKPyVAN diagnosis	4.4 (2.3–6.1)	4.6 (4.6–6.3)	0.55
Antimetabolite dose at diagnosis in mg, mean (SD)	1735 (350)	1705 (364)	0.83
Indication biopsy, n (%)	12 (100)	11 (61)	0.02
Treatment of concurrent rejection, n (%)			0.18^a^
No rejection treatment	6 (50.0)	15 (83.3)	
Steroid bolus	4 (33.3)	2 (11.1)	
ATG	1 (8.3)	1 (5.6)	
Apheresis	1 (8.3)		
Interstitial inflammation, Banff (i) score, n (%)			0.81
Banff i0	2 (16.7)	3 (18.8)	
Banff i1	5 (41.7)	9 (56.3)	
Banff i2	4 (33.3)	3 (18.8)	
Banff i3	1 (8.3)	1 (6.3)	
Tubulitis, Banff (t) Score, n (%)			0.31
Banff t0	3 (27.3)	4 (25.0)	
Banff t1	0 (0.0)	3 (18.8)	
Banff t2	6 (54.5)	6 (37.5)	
Banff t3	2 (18.2)	3 (18.8)	
Allograft loss within the first year after BKPyVAN biopsy, n (%)	2 (16.7)	2 (11)	0.99
Valganciclovir prophylaxis, n (%)	10 (83)	13 (72)	0.67

ATG, antithymocyte globulin; Banff I, interstitial inflammation score; Banff t, tubulitis score; BKPyV, BK polyomavirus; BKPyVAN, BK polyomavirus–associated nephropathy; DNAemia, viral DNA detected in blood; IQR, interquartile range; KTX, kidney transplant.

P = 0.1 for any therapy vs. no therapy.

This table summarizes key continuous and categorical parameters stratified by viral clearance status at 12 months postdiagnosis.

a Across 3 therapy variables.

### ISGs and Absolute BKPyV-DNAemia

**Figure 2:**
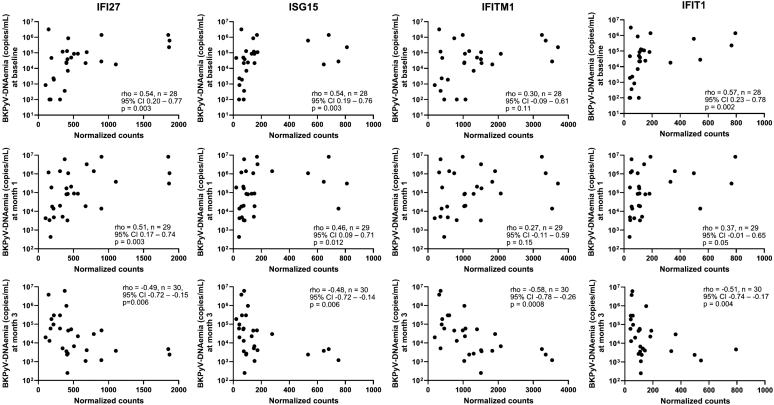
Correlation analysis of gene expression of 4 relevant genes and viral dynamics during the first 3 months after diagnosis. The x-axis represents the expression level of each gene, y-axis represents BKPyV-DNAemia at baseline, 1 and 3 months after the diagnosis of BKPyVAN (copies/ml). BKPyVAN, BK polyomavirus-associated nephropathy; CI, confidence interval; *IFI27*, IFN alpha–inducible protein 27; *IFIT1*, IFN-induced protein with tetratricopeptide repeats 1; *IFITM1*, IFN-induced transmembrane protein 1; *ISG15*, interferon-stimulated gene 15.

At baseline, the expression levels of *ISG15*, *IFI27*, and *IFIT1* but not of *IFITM1* correlated significantly with BKPyV-DNAemia (rho = 0.54, *P* = 0.003; rho = 0.54, *P* = 0.003; rho = 0.57, *P* = 0.002; and rho = 0.30, *P* = 0.11, respectively). One month later, the correlations remained positive but decreased in magnitude (Figure 2). Conversely, by month 3 after index biopsy, all 4 ISGs exhibited significant negative correlations with BKPyV-DNAemia: *IFI27* and *IFIT1* rho = −0.49 and −0.51, both *P* < 0.005, *ISG15* rho = −0.48, *P* = 0.006, and *IFITM1* rho = −0.58, *P* = 0.0008; Figure 2).

### ISGs and BKPyV-DNAemia Decline

**Figure 3:**
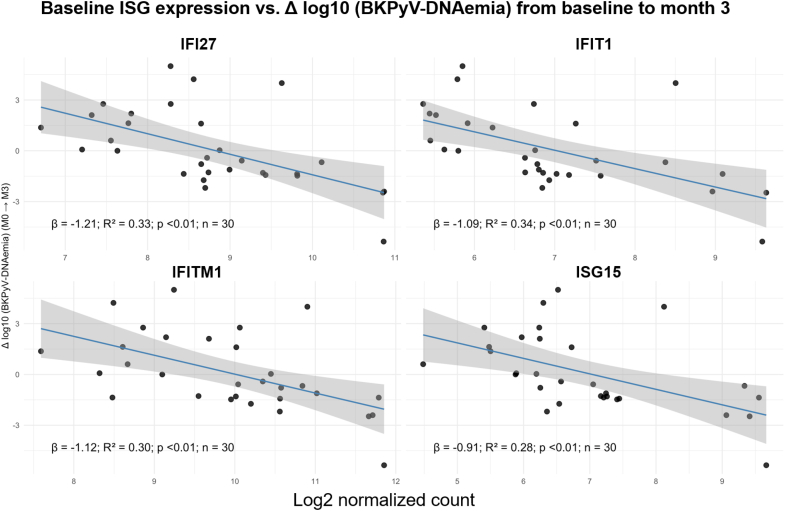
Baseline intragraft interferon-stimulated gene (ISG) expression and subsequent change in BKPyV-DNAemia between baseline and month 3. Each panel depicts the relationship between baseline log2-transformed expression of the 4 assessed ISGs (*IFI27, IFIT1, IFITM1, ISG15*) and the plasma BKPyV-DNAemia dynamics (Δlog_10_, month 0 → month 3). Every dot represents 1 kidney-transplant recipient; the blue line shows the fitted linear regression with its 95% confidence interval (grey shading). Across all 4 genes, the regression slopes are negative, indicating that higher baseline ISG expression was associated with a larger decline in viral load over the following 3 months. Displayed within each panel are the respective regression coefficients (β), determination coefficients (R^2^), and *P*-values, illustrating that the associations were consistent and statistically significant for all 4 ISGs. *IFI27*, IFN alpha–inducible protein 27; *IFIT1*, IFN-induced protein with tetratricopeptide repeats 1; *IFITM1*, IFN-induced transmembrane protein 1; *ISG15*, interferon-stimulated gene 15.

When assessing the DNAemia decline over time, no significant associations were observed between baseline ISGs expression and early DNAemia clearance from baseline to month 1 (all *P* > 0.4). In contrast, higher baseline expression of *IFI27, IFIT1, IFITM1,* and *ISG15* was significantly associated with a greater decline in BKPyV-DNAemia between baseline and month 3 (β range =−0.91 to −1.21; *R*^*2*^ = 0.28–0.34; all *P* < 0.01) (Figure 3). When evaluating the intermediate interval from month 1 to month 3, these associations remained robust, with the strongest effects observed for *IFI27* (β = −1.21, *R*^*2*^ = 0.33, *P* < 0.01), followed by *IFIT1, IFITM1,* and *ISG15* (β range = −0.91 to −1.12; *R*^*2*^ = 0.28–0.34; all *P* < 0.01, Supplementary Figure S2).

Mixed-effects modeling demonstrated significant gene-time interactions for all 4 ISGs (*P* < 0.01), indicating that higher baseline expression was associated with a faster decline in plasma viral load. The effect was consistent from baseline to month 3 and remained pronounced from month 1 to month 3 (β range = −0.30 to −0.54). In Supplementary Figure S3, we show modeled BKPyV-DNAemia trajectories for patients with high (≥ 75th percentile, orange) versus low (≤ 25th percentile, green) baseline ISG expression, whereas in Supplementary Figure S4, we show the individual DNAemia course for patients with high or low baseline ISG expression presented as spaghetti plot. In contrast, prebiopsy changes in BKPyV-DNAemia from month −1 to index-biopsy showed no strong or consistent association with baseline intragraft ISG expression (all adjusted *P* > 0.08; Supplementary Figure S5).

### ROC Analysis for BKPyV-DNAemia and Cross-Validation

**Figure 4:**
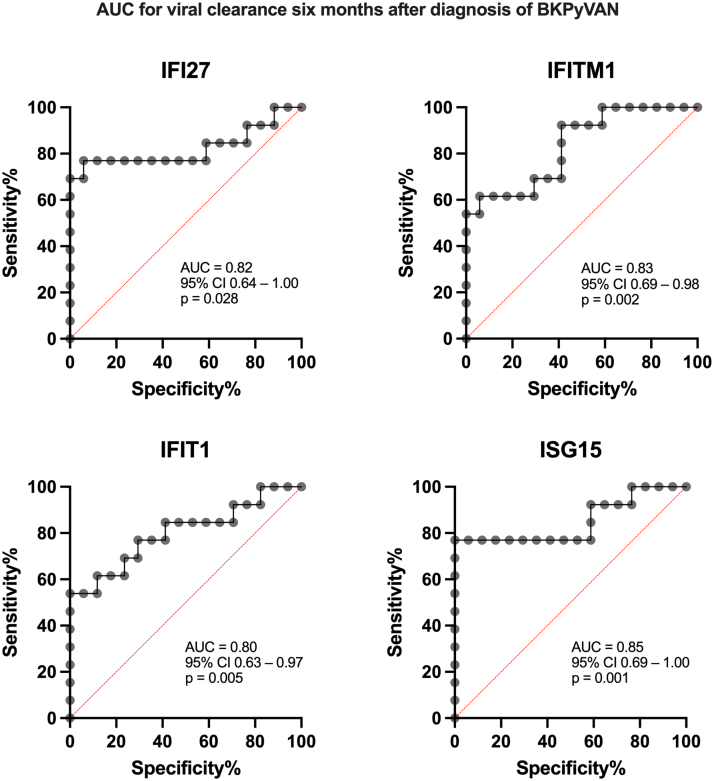
Receiver operating characteristic curves demonstrating the predictive performance of *IFI27, IFITM1, IFIT1*, and *ISG15* with respect to BKPyV-DNAemia clearance at 6 months after the diagnosis of BKPyVAN. Each panel shows sensitivity and specificity, with the corresponding AUC, 95% CI, and *P*-value indicated. AUC, area under the curve; BKPyVAN, BK polyomavirus-associated nephropathy; CI, confidence interval; *IFI27,* IFN alpha–inducible protein 27; *IFIT1*, IFN-induced protein with tetratricopeptide repeats 1; *IFITM1*, IFN-induced transmembrane protein 1; *ISG15*, interferon-stimulated gene 15.

**Figure 5:**
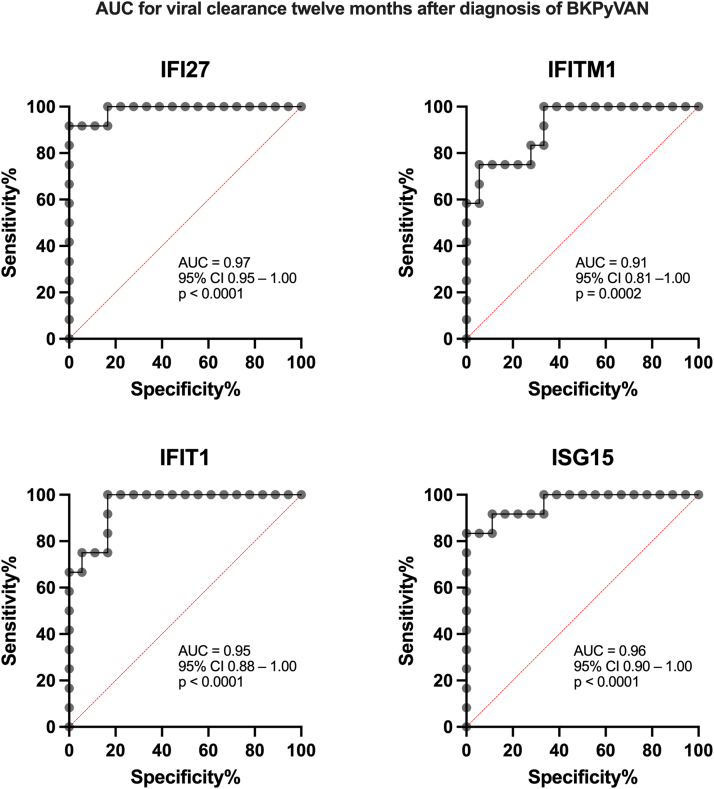
Receiver operating characteristic curves demonstrating the predictive performance of *IFI27, IFITM1, IFIT1*, and *ISG15* with respect to BKPyV-DNAemia clearance at 12 months after the diagnosis of BKPyVAN. Each panel shows sensitivity and specificity, with the corresponding AUC, 95% CI, and *P*-value indicated. AUC, area under the curve; BKPyVAN, BK polyomavirus-associated nephropathy; CI, confidence interval; BKPyVAN, BK polyomavirus-associated nephropathy; *IFI27,* IFN alpha–inducible protein 27; IFIT1, IFN-induced protein with tetratricopeptide repeats 1; IFITM1, IFN-induced transmembrane protein 1; ISG15, interferon-stimulated gene 15.

ROC analysis confirmed significant associations between the 4 genes and viral clearance 6 and 12 months after BKPyVAN diagnosis. To predict virus clearance after 6 months, *ISG15, IFI27, IFITM1*, and *IFIT1* reached AUC values of 0.85, 0.82, 0.83, and 0.80, respectively (*P* < 0.05 for all genes, Figure 4). The genes maintained high predictive relevance for the end point virus clearance after 12 months: AUCs of 0.97, 0.96, 0.95, and 0.91 (for *IFI27, ISG15, IFIT1* and *IFITM1*, respectively; *P* < 0.001 for all genes, Figure 5). For the cross-validation, we performed a single-gene logistic model for viral clearance 6 months after diagnosis using a leave-one-out cross-validation model. The model showed substantial classification performance, with AUC values of 0.79 for both *ISG15* and *IFI27*, 0.78 for *IFITM1*, and 0.71 for *IFIT1* for clearance after 6 months. Discrimination for viral clearance after 12 months was even higher: 0.89 for *ISG15*, 0.91 for *IFI27* and *IFIT1*, and 0.90 for *IFITM1*.

### Gene Expression and Correlation With Graft Function

**Figure 6:**
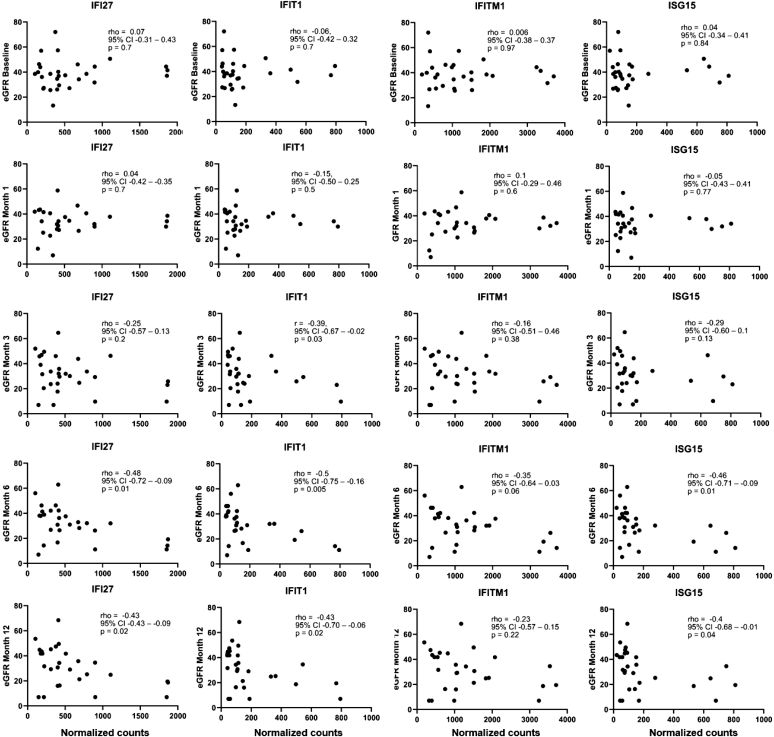
Correlation pattern between each gene (*ISG15, IFI27, IFITM1, IFIT1*) and the eGFR (based on CKD-EPI) measured at baseline, 1, 3, 6, and 12 months after the diagnosis of definite BKPyVAN. Scatterplots showing Spearman’s correlation between expression levels of *ISG15, IFI27, IFITM1*, and *IFIT1* with eGFR (ml/min per 1.73 m^2^) at baseline, 1, 3, 6, and 12 months after diagnosis of definite BKPyVAN. Correlation coefficients, 95% CIs, and *P*-values are shown in each panel. BKPyVAN, BK polyomavirus-associated nephropathy; CI, confidence interval; CKD-EPI, Chronic Kidney Disease-Epidemiology Collaboration; eGFR, estimated glomerular filtration rate; BKPyVAN, BK polyomavirus-associated nephropathy; *IFI27*, IFN alpha–inducible protein 27; *IFIT1*, IFN-induced protein with tetratricopeptide repeats 1; *IFITM1*, IFN-induced transmembrane protein 1; *ISG15*, interferon-stimulated gene 15.

eGFR declined progressively both before and after BKPyVAN diagnosis. At the time of index biopsy, the median eGFR was 38.5 (IQR: 30.6–44.4) ml/min per 1.73 m^2^. Median eGFR then decreased to 34.2 (IQR: 27.9–40.6) ml/min per 1.73 m^2^ at month 1 and to 31.6 (IQR 23.3–44.8) ml/min per 1.73 m^2^ at month 3 after BKPyVAN diagnosis. At month 6, eGFR stabilized at 32.0 (IQR 21.0–40.8) ml/min per 1.73 m^2^. None of the analyzed ISGs showed a significant association with eGFR at the time of BKPyVAN diagnosis (Figure 6). In contrast, higher ISG expression at baseline correlated significantly with lower eGFR at month 6 (*ISG15*: rho = −0.46, *P* = 0.01; *IFI27*: r = −0.48, *P* = 0.01; and *IFIT1*: rho = −0.51, *P* = 0.005) except for *IFITM1* (rho = −0.35, *P* = 0.06). Similar findings were obtained for month 12 (*ISG15*: rho = −0.39, *P* = 0.04; *IFI27*: rho = −0.43, *P* = 0.02; *IFIT1*: rho = −0.43, *P* = 0.02; and *IFITM1*: rho = −0.23, *P* = 0.22). In the extended 24-month follow-up period (data from 26 patients), none of the examined genes showed a statistically significant association with eGFR. ISG-related gene expressions did not differ significantly between patients with and without graft loss during follow-up (Supplementary Table S4). Furthermore, we did not find a significant correlation between ISGs expression levels and recipient age in years: *IFI27*: rho = −0.01, *P* = 0.96; *ISG15*: rho = 0.01, *P* = 0.99; *IFITM1*: rho = −0.09, *P* = 0.65; and *IFIT1*: rho = 0.072, *P* = 0.71.

### Sensitivity Analyses

Given the clinical heterogeneity of the cohort, additional sensitivity analyses were performed to assess robustness of the intragraft transcriptomic findings. Therefore, all inferential analyses were restricted to indication biopsies only. Within this subgroup, differential expression analyses were repeated with adjustment for rejection therapy exposure and BKPyVAN disease severity (PVN score).

In these adjusted analyses, after false discovery rate correction, the core ISG signature remained preserved. At 6 months, *ISG15* (logFC = 2.10, *P* = 0.03) remained significantly associated with virologic outcome, whereas *IFI27* (logFC = 1.49, *P* = 0.08) and *IFIT1* (logFC = 1.69, adjusted *P* = 0.11) showed consistent effect directions despite reduced statistical power. At 12 months, the association was more pronounced, with *IFI27* (logFC = 1.68, *P* = 0.006), *ISG15* (logFC = 2.13, *P* = 0.007), *IFIT1* (logFC = 1.90, *P* = 0.009), and *IFITM1* (logFC = 1.39, *P* = 0.03) all remaining significant after adjustment.

These results are summarized in Supplementary Figure S6, which presents adjusted volcano plots for 6- and 12-month outcomes. Overall, adjustment for rejection treatment and disease severity did not attenuate the intragraft ISG signal, supporting its robustness across clinically relevant sources of heterogeneity.

### Gene Expression and Correlation With Banff Single Lesion Scores

ISG gene expression did not correlate with inflammation (Banff i): *IFI27*: rho = 0.27, *IFIT1*: rho = 0.07, *IFITM1*: rho = 0.21, *ISG15*: rho = 0.22, (*P* > 0.05 for all), or with tubulitis (Banff t): *IFI27*: rho = 0.16, *IFIT1*: rho < 0.01, *IFITM1*: rho = 0.21, *ISG15*: rho = 0.06, (*P* > 0.05 for all). We did not observe a significant association with the PVN score: *IFI27*: rho = 0.14, *P* = 0.46; *IFIT1*: rho = 0.06, *P* = 0.76; *IFITM1*: rho = 0.009, *P* = 0.96; *ISG15*: rho = 0.12, *P* = 0.52 (Supplementary Figure S7). With regard to correlation with the histologic severity according to AST-IDCOP classification, *IFITM1* showed a moderate positive association (rho = 0.43, *P* = 0.02), whereas *IFI27* (rho = 0.33, *P* = 0.08), *IFIT1* (rho = 0.26, *P* = 0.16), and *ISG15* (rho = 0.18, *P* = 0.35) did not reach statistical significance.

## Discussion

The aim of our study was to identify intragraft gene expression patterns predictive of BKPyV-DNAemia clearance in kidney transplant recipients with biopsy-proven BKPyVAN. As a main result, we showed that regulation of ISG was associated with successful BKPyV clearance and that commercially available RNA analysis platforms can be used to depict those processes in clinical routine. Notably, successful DNAemia clearance and high ISG expression were associated with temporarily worse graft function, possibly as a result of collateral allograft injury because of a more intensive antiviral immune response.^25^^,^^26^ Our findings provide a first indication that intragraft gene expression profiling may offer additional valuable insights into antiviral immune response monitoring in patients with BKPyVAN. Importantly, apart from IFN-associated genes, none of the other >400 tested candidate genes showed similar significant associations.

Type I IFN signaling is a crucial component of the innate immune system that responds to viral pathogens.^27^, ^28^, ^29^, ^30^ Infected cells secrete type I IFNs to transform into antimicrobial states, modulate and balance innate immune responses, and activate adaptive immunity.^30^ Those mechanisms are mediated by ISG expression. We observed that 4 members of this group (*IFI27, ISG15, IFIT1,* and *IFITM1*) are significantly upregulated in patients with BKPyVAN who achieved BKPyV-DNAemia clearance. Associations between these genes and other viral pathogens have been described before: *IFI27* upregulation has been observed in HIV, hepatitis C, respiratory syncytial virus, and SARS-CoV-2 infections, serving primarily as a suppressor of viral replication and a modulator of apoptosis.^31^, ^32^, ^33^, ^34^, ^35^ In SARS-CoV-2 and respiratory syncytial virus, *IFI27* expression in the respiratory tract correlated with higher viral loads, and a more severe disease course, supporting its role as an indicator of antiviral immune responses.^32^^,^^36^ Similar to *IFI27*, previous studies reported that upregulated *IFIT1* and *ISG15* expression is associated with responses to various viral infections, including influenza and coronavirus.^37^, ^38^, ^39^, ^40^, ^41^

BKPyV infection of primary human renal proximal tubular epithelial cells revealed that BKPyV replication is sensitive to type I IFN and effective downregulation of innate immune responses is mediated by the BKPyV agnoprotein to ensure high-level replication.^42^ Endothelial cells exposed to BKPyV mount a pronounced type I IFN response, including ISG expression.^43^ IFN-γ upregulated MHC-I and MHC-II and treatment with IFN-γ inhibited viral protein expression.^44^^,^^45^ However, IFN-γ has been linked to upregulation of PD-1L on renal proximal tubular cells, the ligand activating the exhaustion marker PD-1.^46^^,^^47^ Zareei *et al.*^48^ reported that IFN-γ mRNA expression in blood was significantly higher in patients with BKPyVAN (58.5-fold) than in noninfected patients and healthy controls. Similar to our findings for *ISG15* and *IFI27*, IFN-γ levels correlated strongly with BKPyV-DNAemia levels. Our results complement these previous experimental findings by showing that ISG upregulation is detectable in kidney allograft biopsy analyzed with a commercially available gene expression platform and antivirus immune responses may be predicted.

The observed inverse correlation between ISG expression levels and short-term graft function decline may suggest a dual effect of ISG-associated immune responses. Elevated expression of these genes appears to aid in clearing the virus. In contrast, the heightened inflammation that successfully suppresses viral replication can cause collateral injury to the kidney tissue.^25^^,^^26^ Similarly, Drachenberg *et al.*^4^ observed that every BKPyV-DNAemia decrease ≥ 1 logarithm correlated with worse graft function. This immune reconstitution–associated impaired allograft function may be caused by a “beneficial” BKV-specific immune response and needs to be differentiated from persistent BKPyVAN or rejection.^3^ Indeed, we observed that temporary graft dysfunction attenuated after 24 months, possibly because antiviral immune responses subsided.^49^ In contrast to expectations, the severity of inflammatory lesions on light microscopy did not correlate with higher ISG expression levels. Further, viral clearance success rates correlated with ISG expressions but not with Banff lesion scores, similar to earlier reports.^4^ Both findings may support the implementation of gene expression assessments as a complementary diagnostic tool in BKPyVAN management. *IFI27* expression was already suggested as a candidate gene to differentiate immune responses in other renal injury patterns. Adam *et al.*^50^ recently compared gene expression profiles between native kidneys with immune-checkpoint inhibitor–associated acute interstitial nephritis and allografts with TCMR. In their study, majority of genes did not differ; however, *IFI27* was significantly more expressed in TCMR samples (fold change: 2.2) and was therefore suggested as a new biomarker. Notably, 10 biopsies with BKPyVAN were included in a validation cohort. In those, *IFI27* expression was significantly lower than in TCMRs. This convergence may suggest 2 hypotheses. First, despite differing clinical contexts, allo- and antiviral immune responses may share common molecular pathways; however, the amplitude and the timing of the course of infection differ. Second, if sequential testing indicates persistently high ISG expression despite BKPyV-DNAemia clearance, it may help to identify patients at high risk of subsequent rejection episodes. Our results support further studies in larger cohorts, and if confirmed, prospective interventional trials.

Our analysis did not include noninvasive BKPyV immune monitoring approaches, particularly BKPyV-specific T-cell assays, which may play a central role in viral control.^19^ However, these peripheral blood–based measurements primarily reflect systemic immune competence and do not directly inform about local immune processes within the renal allograft. The NanoString platform was selected for its suitability for retrospective analysis of archived biopsy tissue, whereas BKPyV-specific T-cell assays typically require prospective sampling. Prospective studies integrating intragraft and noninvasive immune monitoring will be required to define their combined clinical utility.

Our study, though providing valuable insights into gene expression profiles and clinical outcomes in polyomavirus nephropathy, is not without limitations. Accordingly, the observed associations should be interpreted as correlative, and higher ISG expression may reflect stronger immune responses driven by higher initial viral burden rather than a causal mechanism of viral clearance. In addition, the applied gene set did not include all virus-related genes, such as the previously suggested Vp2 encoding gene (*VP2)*, as well as certain recently identified transcriptome signatures, associated with BKPyV-induced mitochondrial stress patterns.^51^^,^^52^ We used 2 different NanoString panels and despite restriction to overlapping genes (Supplementary Table S5) and the high reproducibility of the nCounter platform, we cannot eliminate potential panel-related effects with full certainty.^53^ Our study is further limited by the missing assessment of *de novo* donor-specific antibodies after transplantation, owing to the lack of standardized donor-specific antibody monitoring in earlier transplant periods. The retrospective nature of the cohort introduces unavoidable heterogeneity in biopsy timing relative to BKPyV disease evolution. Accordingly, the index biopsy was treated as the only consistent temporal reference point, and secondary sensitivity analyses were used to exclude major imbalances. Ultimately, genetic predispositions may additionally modulate IFN-mediated antiviral defense mechanisms; however, testing for their presence was beyond the scope of this analysis.^54^

In conclusion, our findings suggest that intragraft ISG expression is a potential complementary biomarker to monitor antiviral-immune responses associated with viral clearance following BKPyVAN. Increased ISG expression, possibly reflecting immune reconstitution, led to temporarily impairment of graft function, which later stabilized. Further prospective studies are needed to implement ISG measurements as an additional tool to monitor and, if possible, improve individual treatment response and risk assessment in patients with BKPyVAN.

## Disclosure

All the authors declared no competing interests.

## Supplementary Material

Supplementary File (PDF and xlsx) **Supplementary Methods. Supplementary References. Figure S1.** Individual BKPyV-DNAemia and eGFR trajectories. **Figure S2.** Baseline intragraft interferon-stimulated gene (ISG) expression and subsequent change in BKPyV-DNAemia between months 1 and month 3 (addition to Figure 3). **Figure S3.** Mixed effects model trajectories of BKPyV-DNAemia after 3 months. **Figure S4.** Plasma BKPyV-DNAemia trajectories stratified by baseline interferon-stimulated gene (ISG) expression. **Figure S5.** Baseline intragraft interferon-stimulated gene (ISG) expression and subsequent change in BKPyV-DNAemia prior to biopsy. **Figure S6.** Volcano plots showing differential intragraft gene expression associated with virologic outcome at 6 months (panel A) and 12 months (panel B), restricted to indication biopsies and adjusted for rejection therapy exposure and BKPyVAN disease severity (PVN score). **Figure S7.** Boxplot representing ISG expressions (normalized counts) compared between different histological scores (Banff i, Banff t, PVN and AST-IDCOP). **Table S1.** Full list of included genes (PDF). **Table S2.** Distribution of relevant BANFF lesions in the index biopsy. **Table S3.** Differential gene expression analysis results for all genes (xlsx). **Table S4.** Gene expression by graft loss status at 1 year after BKPyVAN diagnosis. **Table S5.** Full list of excluded non overlapping genes (xlsx). STROBE Statement.

## Footnotes

Supplementary File (PDF and xlsx)

**Supplementary Methods.**

**Supplementary References.**
**Figure S1.** Individual BKPyV-DNAemia and eGFR trajectories. **Figure S2.** Baseline intragraft interferon-stimulated gene (ISG) expression and subsequent change in BKPyV-DNAemia between months 1 and month 3 (addition to Figure 3). **Figure S3.** Mixed effects model trajectories of BKPyV-DNAemia after 3 months. **Figure S4.** Plasma BKPyV-DNAemia trajectories stratified by baseline interferon-stimulated gene (ISG) expression. **Figure S5.** Baseline intragraft interferon-stimulated gene (ISG) expression and subsequent change in BKPyV-DNAemia prior to biopsy. **Figure S6.** Volcano plots showing differential intragraft gene expression associated with virologic outcome at 6 months (panel A) and 12 months (panel B), restricted to indication biopsies and adjusted for rejection therapy exposure and BKPyVAN disease severity (PVN score). **Figure S7.** Boxplot representing ISG expressions (normalized counts) compared between different histological scores (Banff i, Banff t, PVN and AST-IDCOP). **Table S1.** Full list of included genes. **Table S2.** Distribution of relevant BANFF lesions in the index biopsy. **Table S3.** Differential gene expression analysis results for all genes (xlsx). **Table S4.** Gene expression by graft loss status at 1 year after BKPyVAN diagnosis. **Table S5.** Full list of excluded non overlapping genes (xlsx). STROBE Statement.
