# Supplementary material for: Interferon-Inducible Gene Upregulation Correlates With Successful Viral Clearance in Patients With BK Polyomavirus-Associated Nephropathy
Source: Kidney Int Rep. 2026 Apr 22;11(7):106551. doi: 10.1016/j.ekir.2026.106551 (PMC13200016; doi:10.1016/j.ekir.2026.106551)
Supplement: Supplementary File (PDF and xlsx) — Supplementary Methods. Supplementary References. Figure S1. Individual BKPyV-DNAemia and eGFR trajectories. Figure S2. Baseline intragraft interferon-stimulated gene (ISG) expression and subsequent change in BKPyV-DNAemia between months 1 and month 3 (addition to Figure 3). Figure S3. Mixed effects model trajectories of BKPyV-DNAemia after 3 months. Figure S4. Plasma BKPyV-DNAemia trajectories stratified by baseline interferon-stimulated gene (ISG) expression. Figure S5. Baseline intragraft interferon-stimulated gene (ISG) expression and subsequent change in BKPyV-DNAemia prior to biopsy. Figure S6. Volcano plots showing differential intragraft gene expression associated with virologic outcome at 6 months (panel A) and 12 months (panel B), restricted to indication biopsies and adjusted for rejection therapy exposure and BKPyVAN disease severity (PVN score). Figure S7. Boxplot representing ISG expressions (normalized counts) compared between different histological scores (Banff i, Banff t, PVN and AST-IDCOP). Table S1. Full list of included genes (PDF). Table S2. Distribution of relevant BANFF lesions in the index biopsy. Table S3. Differential gene expression analysis results for all genes (xlsx). Table S4. Gene expression by graft loss status at 1 year after BKPyVAN diagnosis. Table S5. Full list of excluded non overlapping genes (xlsx). STROBE Statement. [file mmc1.pdf]

Supplementary Material

Supplementary methods

Statistical Tests

Statistical analysis was performed using SPSS Statistics 29 (IBM SPSS Statistics for Mac, IBM Corp., Armonk, N.Y., USA) and R Studio Version 2024.09.1+394 (2024.09.1+394) 2024 by Posit Software, PBC for Mac. Descriptive statistics such as mean ± standard deviation (SD) or median (interquartile range, IQR) were used to summarize continuous variables, while categorical variables were presented as frequencies and percentages.

The analysis of the expression data were conducted using R for Mac with the previously published R Package “nanostringr” (1). Background correction involved subtracting the mean count of negative controls plus one standard deviation. Measured expression values were normalized using the means of the supplied controls and housekeeping genes. After quality control and normalization, each gene’s expression was log2-transformed to stabilize the variance. Differential expression analysis was performed in R using the “limma” package.

For each outcome variable, the gene expression matrix was arranged so that genes were treated as dependent variables in a linear model and the outcome was modeled as a two-level factor in the design matrix. The analysis workflow involved fitting the expression data using the “lmFit” function, followed by empirical Bayes moderation via the “eBayes” function. This approach calculates a moderated t-statistic for each gene, which is more robust than a simple t-test in small-sample contexts. The “topTable” function was then used to extract genes ranked by their moderated t-statistics and to compute raw and adjusted p-values. To control for multiple comparisons, the Benjamini–Hochberg procedure was applied, yielding false discovery rate (FDR)–adjusted p-values. Genes with an adjusted p-value below a defined threshold ($p < 0.01$) were considered differentially expressed. All analyses were carried out in R version 2024.09.1+394, and the package versions used were consistent with the CRAN release at the time of the study.

Secondarily, for significant genes, additional predictive analyses were performed using single-gene logistic regression, and receiver operating characteristic curve analysis was applied to assess discriminative performance. All data handling and transformations, including the log2 conversion of expression values, followed accepted practices for small-sample, high-dimensional datasets. Spearman correlation tests assessed relationships between continuous variables and gene expression levels.

The gene expression patterns were displayed as a volcano plot with appropriate logFC as well as their p-value for FDR. The ROC analysis was used to assess the predictive power of each significant gene for viral clearance after 6 and 12 months. For the Cross-Validation procedure**,** we evaluated our logistic regression models using a leave-one-out cross-validation (LOOCV) approach. The model is trained on the training set and subsequently tested on the held-out subject. This process is repeated for every subject, ensuring that each individual acts as a test case exactly once. Finally, the predictive performance measures (such as the area under the ROC curve, AUC) are averaged across all iterations, providing an overall estimate of how well the model generalizes to unseen data.

To address potential confounding, a range of variables was recorded, including patient age, gender, number of previous transplants, living donor status (yes/no), underlying disease, HLA mismatch, cytomegalovirus (CMV) status, cold ischemia period, and the presence of donor-specific antibodies. Detailed immunosuppressive treatment schedules and dosages, administered both before and during the diagnosis of BKPyVAN, were also collected. Confounders significantly different at the time of diagnosis, typically associated with viral clearance (such as the use of ATG, immunosuppression levels, and rejection after the diagnosis of BKPyVAN), were adjusted using multivariable regression models.

Ethical approval was obtained from the Institutional Review Board of the Medical University of Vienna (reference number: 1543/2018), and the study was conducted in compliance with all applicable ethical standards

Supplementary Tables

Supplementary Table S1. Full list of included genes

ADAMDEC1	CCR5	CDKN1A	CDKN1A	GBP2	IL16	KIR_Activating_Subgroup_2	NFKBIA	SLC11A1	VEGFA
ADORA2A	CCR6	CFB	CFB	GNLY	IL17A	KIR_Inhibiting_Subgroup_1	NKG7	SMAD2	VEGFC
AICDA	CCR7	CFI	CFI	GZMA	IL17F	KIR_Inhibiting_Subgroup_2	NLRC5	SMAD3	ZAP70
AIM2	CD14	CHUK	CHUK	GZMB	IL17RA	KIT	NLRP3	SOCS1	
AIRE	CD160	CLEC4C	CLEC4C	GZMH	IL17RB	KLRB1	NOD1	SP140	
ANKRD22	CD163	CMKLR1	CMKLR1	GZMK	IL18	KLRC1	NOD2	STAT1	
ANXA1	CD19	COL3A1	COL3A1	HAVCR2	IL18BP	KLRD1	NOTCH1	STAT3	
AOAH	CD1D	CRP	CRP	HLAA	IL18RAP	KLRF1	PAX5	STAT4	
APOE	CD2	CSF1	CSF1	HLAB	IL1A	KLRG1	PDCD1	STAT5B	
ARG1	CD207	CSF2	CSF2	HLAC	IL1B	KLRK1	PDCD1LG2	STAT6	
ARG2	CD209	CSF2RB	CSF2RB	HLADMA	IL1R1	LAG3	PDGFRB	SYK	
ATM	CD22	CSF3	CSF3	HLADMB	IL1R2	LAMP1	PECAM1	TANK	
AXL	CD24	CSF3R	CSF3R	HLADPA1	IL1RAP	LCK	PIK3CD	TAP1	
B2M	CD244	CTLA4	CTLA4	HLADPB1	IL1RL1	LCN2	PIK3CG	TAP2	
BASP1	CD247	CTSL	CTSL	HLADQA1	IL1RN	LGALS3	PIN1	TAPBP	
BATF	CD27	CTSS	CTSS	HLADQB1	IL2	LIF	PLAU	TBK1	
BAX	CD274	CTSW	CTSW	HLADRA	IL21	LILRB1	PLAUR	TBX21	
BCL2	CD276	CX3CL1	CX3CL1	HLADRB3	IL21R	LILRB2	POU2AF1	TCF7	
BCL2L1	CD28	CX3CR1	CX3CR1	HLAE	IL22	LTA	PPBP	TFRC	
BCL6	CD34	CXCL10	CXCL10	HLAG	IL23A	LTB	PRF1	TGFB1	
BLK	CD38	CXCL11	CXCL11	HMGB1	IL23R	LTBR	PSEN1	TGFB2	
BLNK	CD3D	CXCL12	CXCL12	HSD11B1	IL27	LTF	PSMB10	THBD	
BST2	CD3E	CXCL13	CXCL13	ICAM1	IL2RA	LY96	PSMB8	THBS1	
BTK	CD3G	CXCL14	CXCL14	ICAM2	IL2RB	MAF	PSMB9	TIGIT	
BTLA	CD4	CXCL16	CXCL16	ICOS	IL2RG	MAP3K1	PSME1	TLR2	

C1QA	CD40	CXCL2	CXCL2	ICOSLG	IL4	MAPK11	PTGS2	TLR3
C1QB	CD40LG	CXCL5	CXCL5	IDO1	IL4R	MAPK14	PTPN7	TLR4
C1S	CD44	CXCL9	CXCL9	IFI27	IL5	MAPK3	PTPRC	TLR5
C3	CD46	CXCR3	CXCR3	IFIT1	IL6	MAPK8	PVR	TLR7
C3AR1	CD47	CXCR4	CXCR4	IFITM1	IL6R	MASP1	REL	TLR8
C5	CD48	CXCR5	CXCR5	IFITM2	IL6ST	MASP2	RELA	TLR9
C9	CD5	CXCR6	CXCR6	IFNA1	IL7	MCAM	RELB	TNF
CASP1	CD55	DEFB1	DEFB1	IFNAR1	IL7R	MEF2C	RORA	TNFAIP3
CASP3	CD58	EBI3	EBI3	IFNAR2	INPP5D	MERTK	RORC	TNFRSF14
CASP8	CD59	EGR1	EGR1	IFNG	IRF1	MICA	RPS6	TNFRSF17
CCL13	CD6	ENG	ENG	IFNGR1	IRF4	MICB	RUNX1	TNFRSF18
CCL15	CD68	EOMES	EOMES	IGF1R	IRF7	MIF	S100A12	TNFRSF1A
CCL18	CD7	FADD	FADD	IGF2R	IRF8	MME	S100A8	TNFRSF1B
CCL19	CD70	FAS	FAS	IGHG1	ISG15	MRC1	S100B	TNFRSF4
CCL2	CD72	FCER1A	FCER1A	IGKC	ISG20	MS4A1	SELE	TNFRSF9
CCL20	CD74	FCER1G	FCER1G	IKBKB	ITGA4	MS4A2	SELL	TNFSF10
CCL21	CD79A	FCGR1A	FCGR1A	IKBKG	ITGAM	MUC1	SELPLG	TNFSF14
CCL22	CD80	FCGR2A	FCGR2A	IL10	ITGAX	MX1	SERPING1	TNFSF18
CCL4	CD81	FCGR2B	FCGR2B	IL10RA	ITGB2	MYD88	SH2D1A	TNFSF4
CCL5	CD83	FLT3	FLT3	IL12A	JAK1	NCAM1	SH2D1B	TNFSF8
CCR1	CD84	FN1	FN1	IL12B	JAK2	NCR1	SIGIRR	TP53
CCR2	CD86	FOS	FOS	IL12RB1	JAK3	NFATC1	SLA	TRAF6
CCR3	CD8A	FOXP3	FOXP3	IL12RB2	KIR3DL1	NFATC2	SLAMF6	TREM1
CCR4	CD8B	FYN	FYN	IL13	KIR3DL2	NFKB1	SLAMF7	TYK2
CDH5	CD96	GATA3	GATA3	IL15	KIR_Activating_Subgroup_1	NFKB2	SLAMF8	VCAM1

Supplementary table S2. Distribution of BANFF Lesions and biopsy adequacy.

**BANFF Lesion**	**Score**	**n (%)**
Interstitial inflammation (i)	0	7 (23.3)
	1	14 (46.7)
	2	7 (23.3)
	3	2 (6.7)
Tubulitis (t)	0	10 (33.3)
	1	3 (10.0)
	2	12 (40.0)
	3	5 (16.7)
Intimal arteritis (v)	0	29 (96.7)
	1	1 (3.3)
Glomerulitis (g)	0	30 (100)
Peritubular capillaritis (ptc)	0	22 (73.3)
	1	4 (13.3)
	2	4 (13.3)
C4d staining*	0	23 (79.3)
	1	1 (3.3)
	2	1 (3.3)
	3	4 (13.3)
BKPyVN severity (PVN Class)	1	3 (10.0)
	2	16 (53.3)
	3	11 (36.6)
**Biopsy adequacy**		
Number of glomeruli, median (IQR)		14 (11–17)
Number of interstitial scars, median (IQR)		1 (0–2)
Number of arteries, median (IQR)		2 (1–2)

* no staining available in one patient

Supplementary table S4. Gene expression by graft loss status at 1 year after BKPyVAN diagnosis

Genes	Graft Loss (n=4)	No Graft Loss (n=26)	p-value
IFI27, median (IQR)	558.6 (164.0–1613.5)	411.7 (287.5–677.8)	>0.99
IFITM1, median (IQR)	689.6 (336.1–2681.1)	1060.5 (560.9–1601.6)	0.39
ISG15, median (IQR)	113.8 (47.3–553.2)	92.8 (73.6–157.5)	0.93
IFIT1, median (IQR)	124.3 (51.1–642.3)	109.8 (56.2–159.8)	0.75

Supplementary Figure S1. Individual BKPyV-DNAemia and eGFR trajectories

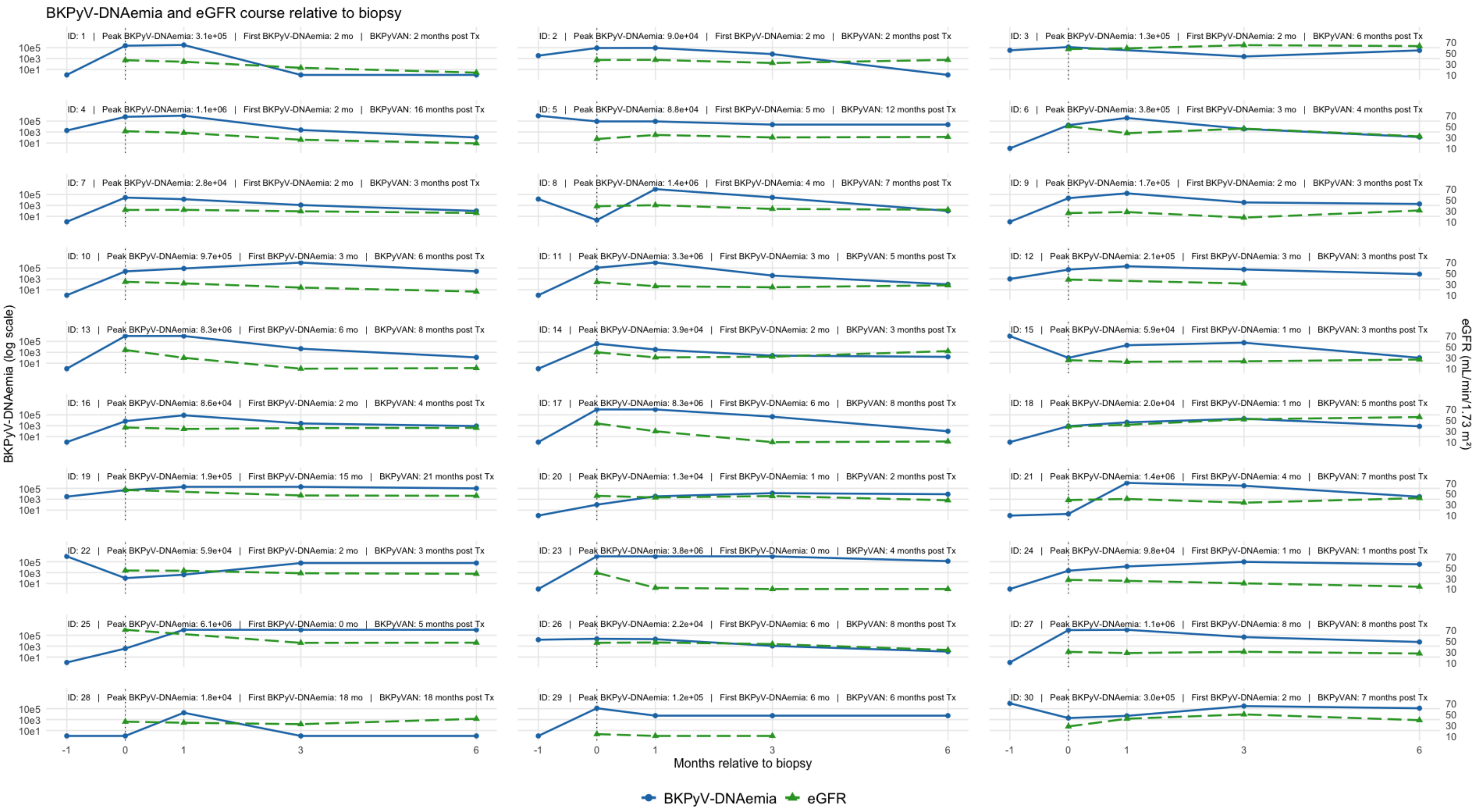

Individual trajectories of BKPyV-DNAemia (incl. one month before the biopsy) and estimated glomerular filtration rate (eGFR) relative to index biopsy (month 0) to month 6. Each panel represents one patient. Blue solid lines and circles indicate plasma viral load displayed on a logarithmic scale (left y-

axis), whereas green dashed lines and triangles represent eGFR on the secondary right y-axis (mL/min/1.73 m²). The dotted vertical line marks the time of biopsy. Top annotations summarize, for each patient, the identifier, peak viremia after transplantation, time to first viremia after transplantation, and time to biopsy-proven BK polyomavirus–associated nephropathy (BKPyVAN) after transplantation. Four patients lost their allograft within the first twelve months after the biopsy

Supplementary Figure S2: Baseline Intragraft interferon-stimulated gene (ISG) expression and subsequent change in BKPyV-DNAemia between months 1 and month 3 (addition to Figure 3).

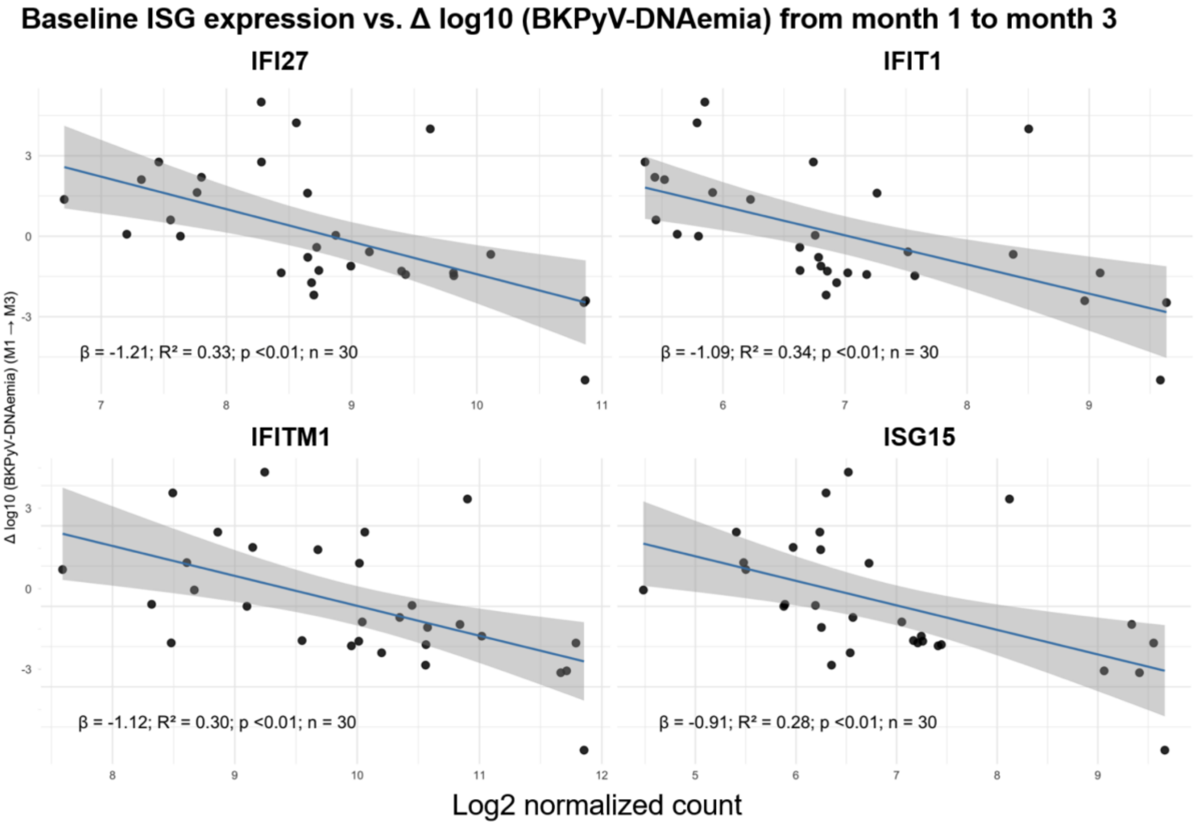

Each panel depicts the relationship between baseline log2-transformed expression of the four assessed ISGs (IFI27, IFIT1, IFITM1, ISG15) and the plasma BKPyV-DNAemia dynamics (Δlog10, month 1 → month 3). Every dot represents one kidney-transplant recipient; the blue line shows the fitted linear regression with its 95 % confidence interval (grey shading). Across all four genes, the regression slopes are negative, indicating that higher baseline ISG expression was associated with a larger decline in viral load over the following two months. Displayed within each panel are the respective regression coefficients (β), determination coefficients (R^2), and p-values, illustrating that the associations were consistent and statistically significant for all four ISGs.

Supplementary Figure S3. Mixed effects model trajectories of BKPyV-DNAemia ofter three months.

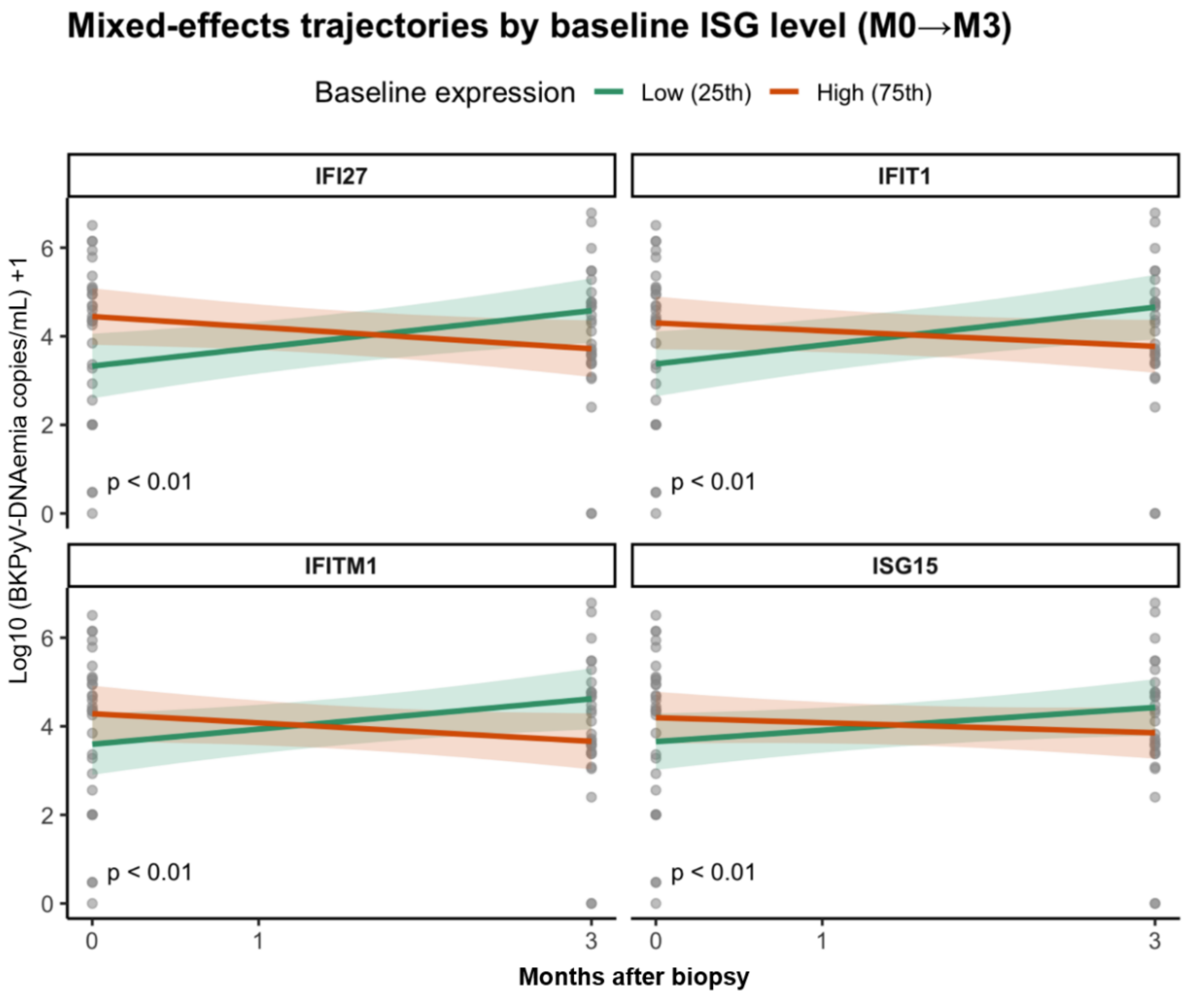

Figure illustrates BKPyV-DNAemia (log10 scale) slope from month 0 to month 3, stratified by baseline interferon-stimulated gene (ISG) expression levels (25th vs. 75th percentile). Patients with higher baseline ISG expression (orange lines) showed a faster decline in viral load over time compared to those with low ISG expression (green lines), across all four genes (IFI27, IFIT1, IFITM1, ISG15).

Supplementary Figure S4. Plasma BKPyV DNAemia trajectories stratified by baseline interferon-stimulated gene (ISG) expression.

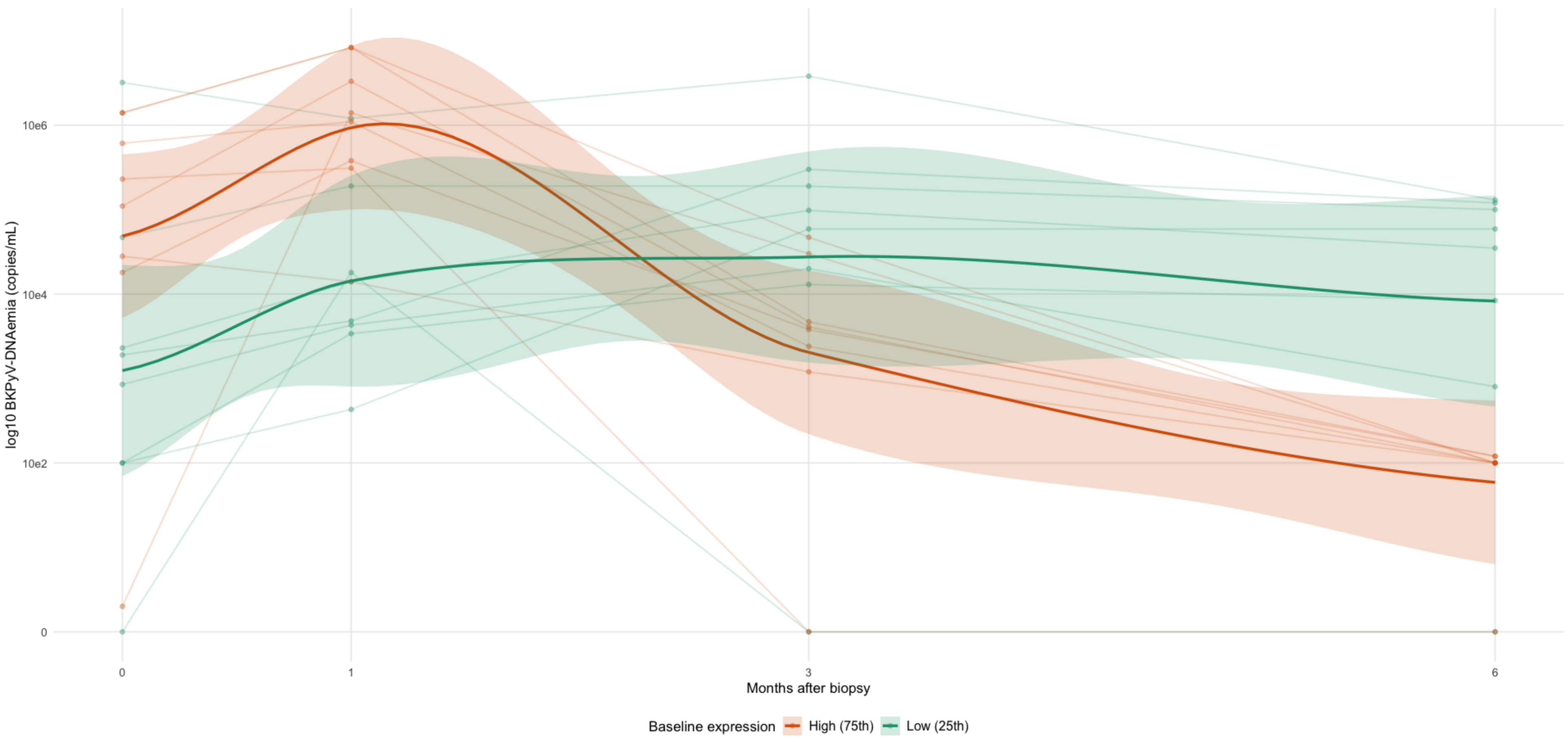

Individual patient trajectories of plasma BKPyV DNAemia (log10 copies/mL) from biopsy (month 0) to month 6 are shown as semi-transparent lines. Patients were stratified into ISG-low (≤25th percentile, green) and ISG-high (≥75th percentile, orange) groups based on a composite ISG score derived from IFI27, IFIT1, IFITM1, and ISG15 expression at baseline. Solid lines represent group-level smoothed trajectories with shaded 95% confidence intervals.

Supplementary Figure S5: Baseline intragraft interferon-stimulated gene (ISG) expression and subsequent change in BKPyV-DNAemia prior to biopsy.

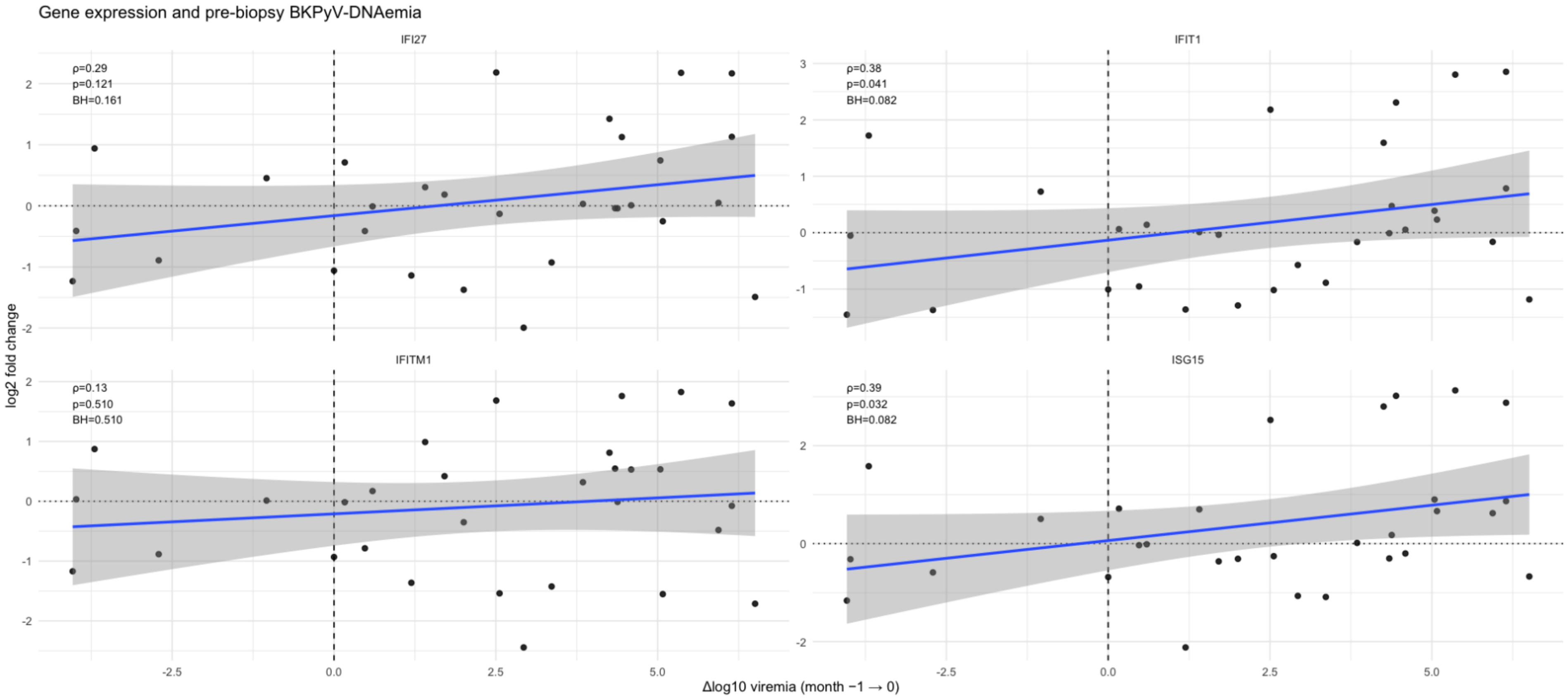

Each panel depicts the relationship between baseline log2-transformed expression of the four assessed ISGs (IFI27, IFIT1, IFITM1, ISG15) and the plasma BKPyV-DNAemia dynamics (Δlog10, month -1 → biopsy). Every dot represents one kidney-transplant recipient; the blue line shows the fitted linear regression with its 95 % confidence interval (grey shading). No significant associations could be found regarding BKPyV-DNAemia changes prior to biopsy and gene expression in the biopsy.

Supplementary Figure S6. Volcano plots showing differential intragraft gene expression associated with virologic outcome at 6 months (panel A) and 12 months (panel B), restricted to indication biopsies and adjusted for rejection therapy exposure and BKPyVAN disease severity (PVN score).

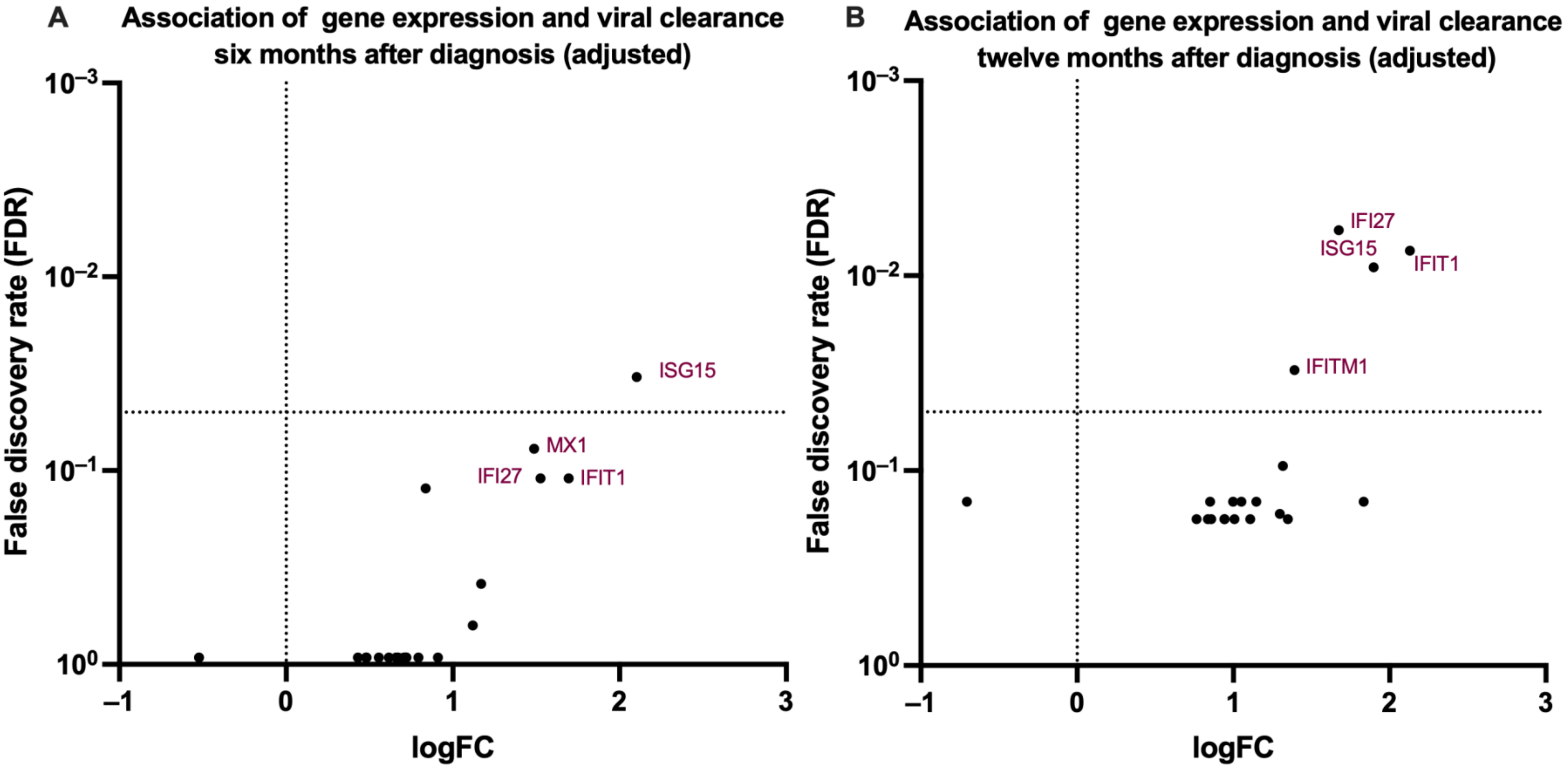

Each dot represents one gene; the x-axis denotes log_2 fold change and the y-axis the –log10 adjusted p value (FDR). Top four genes are annotated. For visualization purposes, only top 20 genes are displayed. Despite adjustment for treatment variability and disease severity, the core ISG signature remained preserved, with strongest associations observed for 12-month virologic outcomes.

Supplementary Figure S7. **Boxplot representing ISG expressions (normalized counts) compared between different histological scores (Banff i, Banff t, PVN and AST-IDCOP).**

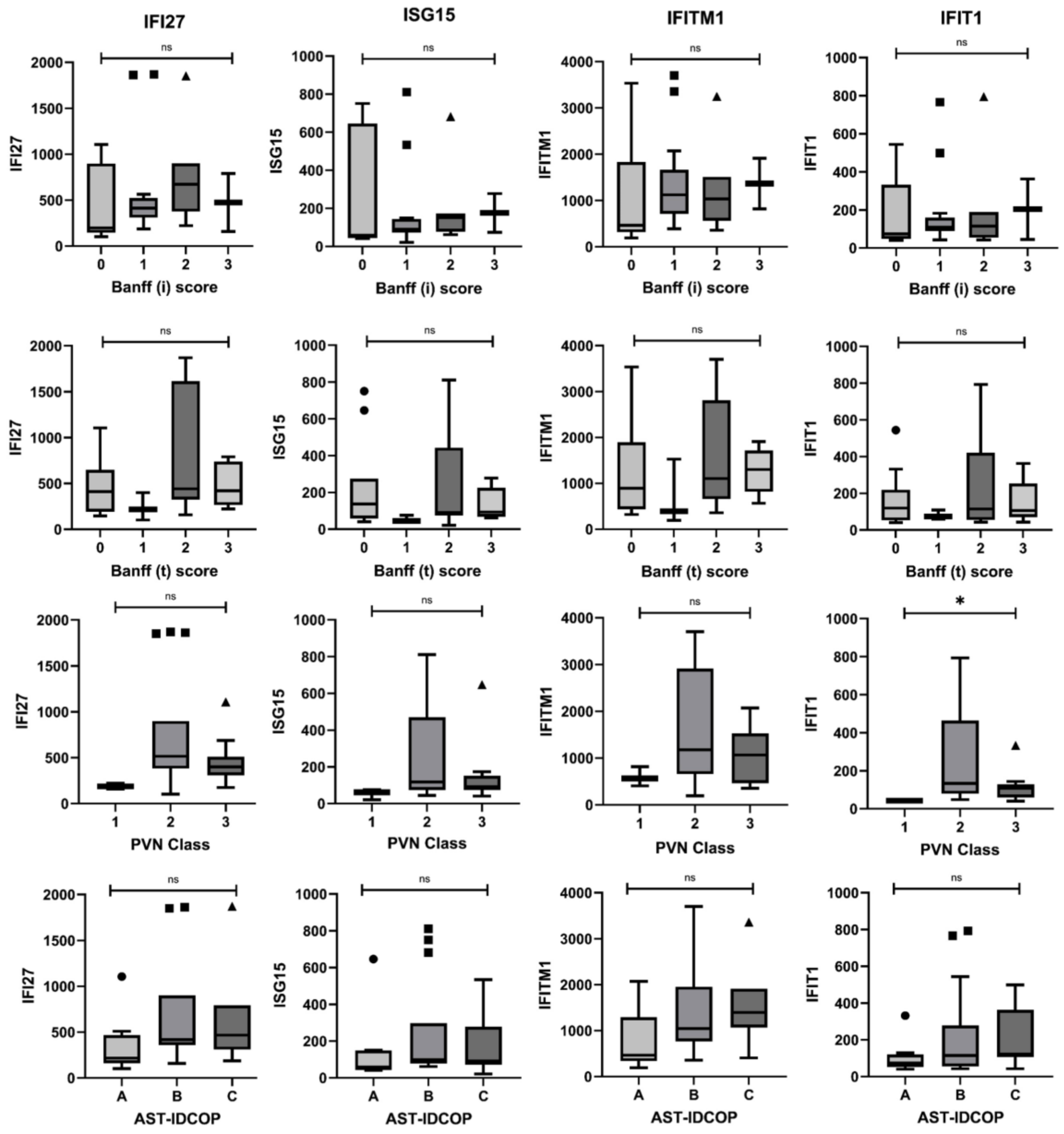

* Indicates a p-value <0.05.

STROBE Statement

	Item No	Recommendation	Page No
**Title and abstract**	1	(*a*) Indicate the study's design with a commonly used term in the title or the abstract	1,3
		(*b*) Provide in the abstract an informative and balanced summary of what was done and what was found	3
**Introduction**			
Background/rationale	2	Explain the scientific background and rationale for the investigation being reported	5
Objectives	3	State specific objectives, including any prespecified hypotheses	5
**Methods**			
Study design	4	Present key elements of study design early in the paper	6
Setting	5	Describe the setting, locations, and relevant dates, including periods of recruitment, exposure, follow-up, and data collection	6
Participants	6	(*a*) *Cohort study*—Give the eligibility criteria, and the sources and methods of selection of participants. Describe methods of follow-up<br>*Case-control study*—Give the eligibility criteria, and the sources and methods of case ascertainment and control selection. Give the rationale for the choice of cases and controls<br>*Cross-sectional study*—Give the eligibility criteria, and the sources and methods of selection of participants	6
		(*b*) *Cohort study*—For matched studies, give matching criteria and number of exposed and unexposed<br>*Case-control study*—For matched studies, give matching criteria and the number of controls per case	N/A
Variables	7	Clearly define all outcomes, exposures, predictors, potential confounders, and effect modifiers. Give diagnostic criteria, if applicable	8,9, Supp.Methods
Data sources/ measurement	8*	For each variable of interest, give sources of data and details of methods of assessment (measurement). Describe comparability of assessment methods if there is more than one group	*9*
Bias	9	Describe any efforts to address potential sources of bias	9

Study size	10	Explain how the study size was arrived at	6,7
Quantitative variables	11	Explain how quantitative variables were handled in the analyses. If applicable, describe which groupings were chosen and why	
Statistical methods	12	(*a*) Describe all statistical methods, including those used to control for confounding	8
		(*b*) Describe any methods used to examine subgroups and interactions	N/A
		(*c*) Explain how missing data were addressed	9
		(*d*) *Cohort study*—If applicable, explain how loss to follow-up was addressed<br>*Case-control study*—If applicable, explain how matching of cases and controls was addressed<br>*Cross-sectional study*—If applicable, describe analytical methods taking account of sampling strategy	N/A
		(*e*) Describe any sensitivity analyses	N/A

Continued on next page

**Results**			
Participants	13*	(a) Report numbers of individuals at each stage of study—eg numbers potentially eligible, examined for eligibility, confirmed eligible, included in the study, completing follow-up, and analysed	10
		(b) Give reasons for non-participation at each stage	N/A
		(c) Consider use of a flow diagram	N/A
Descriptive data	14*	(a) Give characteristics of study participants (eg demographic, clinical, social) and information on exposures and potential confounders	10
		(b) Indicate number of participants with missing data for each variable of interest	11
		(c) *Cohort study*—Summarise follow-up time (eg, average and total amount)	11
Outcome data	15*	*Cohort study*—Report numbers of outcome events or summary measures over time	*11*
		Case-control study—Report numbers in each exposure category, or summary measures of exposure	N/A
		Cross-sectional study—Report numbers of outcome events or summary measures	N/A
Main results	16	(*a*) Give unadjusted estimates and, if applicable, confounder-adjusted estimates and their precision (eg, 95% confidence interval). Make clear which confounders were adjusted for and why they were included	12,13
		(*b*) Report category boundaries when continuous variables were categorized	12/13
		(*c*) If relevant, consider translating estimates of relative risk into absolute risk for a meaningful time period	N/A
Other analyses	17	Report other analyses done—eg analyses of subgroups and interactions, and sensitivity analyses	12/13
**Discussion**			
Key results	18	Summarise key results with reference to study objectives	15
Limitations	19	Discuss limitations of the study, taking into account sources of potential bias or imprecision. Discuss both direction and magnitude of any potential bias	17
Interpretation	20	Give a cautious overall interpretation of results considering objectives, limitations, multiplicity of analyses, results from similar studies, and other relevant evidence	16
Generalisability	21	Discuss the generalisability (external validity) of the study results	18,19
**Other information**			
Funding	22	Give the source of funding and the role of the funders for the present study and, if applicable, for the original study on which the present article is based	20

*Give information separately for cases and controls in case-control studies and, if applicable, for exposed and unexposed groups in cohort and cross-sectional studies.

Note: An Explanation and Elaboration article discusses each checklist item and gives methodological background and published examples of transparent reporting. The STROBE checklist is best used in conjunction with this article (freely available on the Web sites of PLoS Medicine at http://www.plosmedicine.org/, Annals of Internal Medicine at http://www.annals.org/, and Epidemiology at http://www.epidem.com/). Information on the STROBE Initiative is available at www.strobe-statement.org.

Supplementary References

S1. Talhouk A, Kommoss S, Mackenzie R, Cheung M, Leung S, Chiu DS, et al. Single-Patient Molecular Testing with NanoString nCounter Data Using a Reference-Based Strategy for Batch Effect Correction. PLOS ONE. 2016;11(4):e0153844.
